# A blistering neonatal nodule: Diagnostic challenges in cutaneous mastocytoma

**DOI:** 10.1016/j.jdcr.2026.08.050

**Published:** 2026-09-04

**Authors:** Sanam Lasi, Marion Leahy, Johny Salazar, Laoise Griffin, Aisling Shaughnessy

**Affiliations:** Department of Dermatology, University Hospital Limerick, Limerick, Ireland

**Keywords:** blistering disorders, cutaneous mastocytosis, Mastocytoma, neonate

## Case description

A solitary well-circumscribed nodule measuring 1.5 × 1.5 cm on the medial aspect of the left knee was noted on a term neonate at birth. It was violaceous in colour, firm, dome-shaped with a smooth surface. The neonate was otherwise well with an uncomplicated perinatal history. Within 48 hours, the surface developed a vesiculobullous appearance on a yellow-brown background (Fig 1). Following bacterial swabs for culture and sensitivity, empiric intravenous antibiotics were initiated, viral PCR swab was taken after deroofing, and systemic antivirals were also commenced as a precaution. C-reactive protein and white cell count was normal as were vital signs. A full skin examination revealed no additional lesions or organomegaly. An urgent dermatology opinion was sought. Due to increasing size, diagnostic uncertainty and following an assessment of risk versus benefit of local anaesthetic, a punch biopsy was performed. Histology demonstrated a diffuse dermal infiltrate of a monotonous population of cells with round-to-ovoid nuclei and conspicuous pale, faintly granular cytoplasm (Figs 2 and 3). Immunohistochemistry demonstrated strong diffuse CD117 (c-KIT) and CD68 positivity and was negative for CD1a (Fig 4).

**Fig 1:**
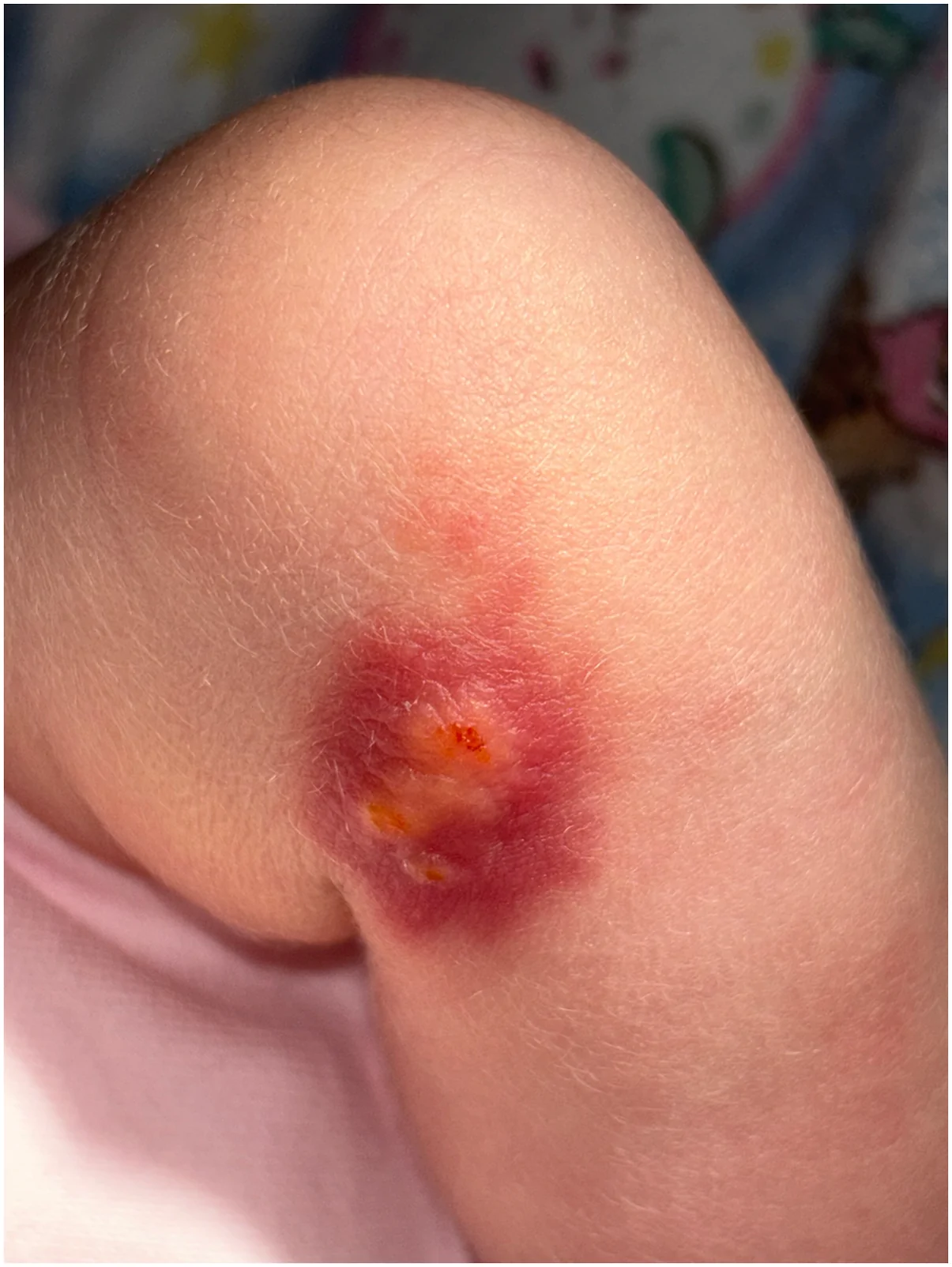
Vesiculobullous change overlying a yellow-brown plaque on the medial left knee at 48 hours of life.

**Fig 2:**
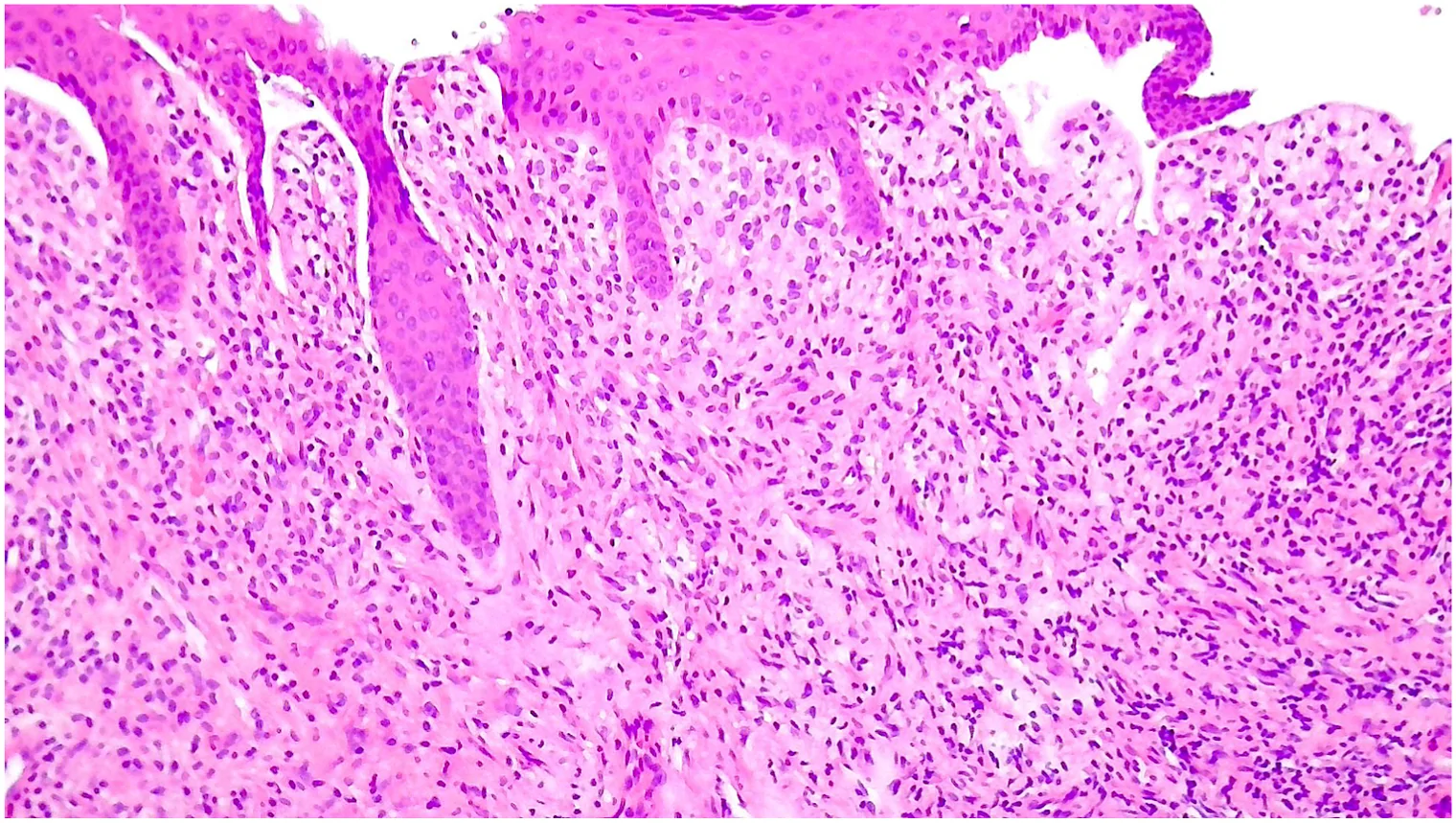
Diffuse dermal infiltrate of monomorphic cells with round-to-ovoid nuclei and pale, faintly granular cytoplasm (×10).

**Fig 3:**
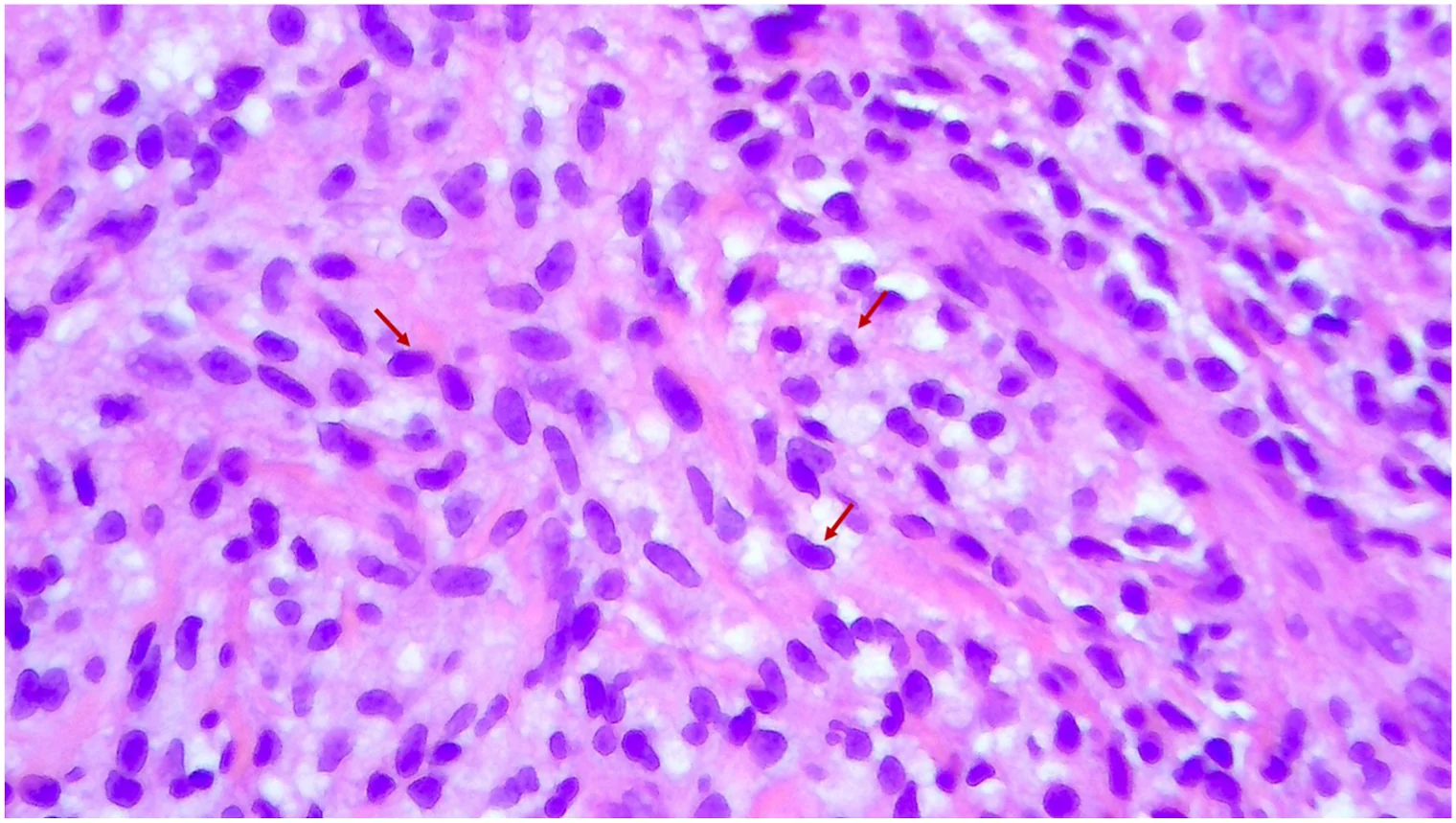
Higher-power view demonstrating dense dermal infiltrates of round cells resembling immature mast cells in the dermis (highlighted) (×40).

**Fig 4:**
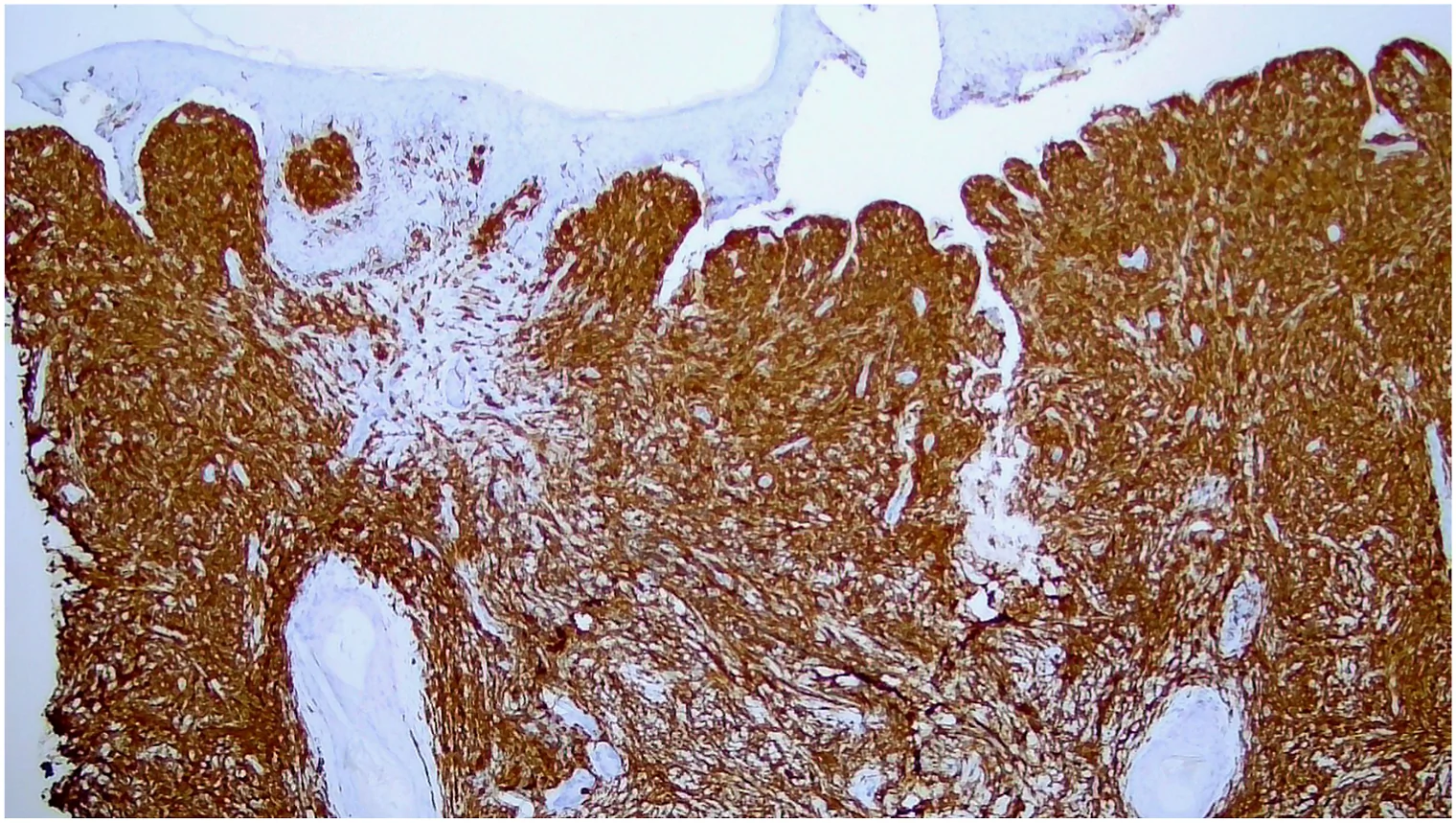
Immunohistochemistry demonstrating strong diffuse CD117 (c-KIT) positivity within the dermal infiltrate (×4).

### Question: What is the most likely diagnosis?

**A.** Bullous impetigo
**B.** Herpes simplex infection
**C.** Langerhans cell histiocytosis
**D.** Cutaneous mastocytoma
**E.** Juvenile xanthogranuloma

## Answer and discussion

**Correct answer is D. Cutaneous mastocytoma.**

Cutaneous mastocytoma typically presents as a solitary lesion at birth or in early infancy.^1^ Blistering may occur following mechanical irritation, reflecting mast cell degranulation, a hallmark feature of cutaneous mastocytosis.^1^^,^^2^ This likely contributed to the vesicular appearance in our case; however, Darier sign was not deliberately elicited during examination. In neonates, bullous change can be more pronounced and may mimic infection. Isolated mastocytoma is usually asymptomatic, although mild localized pruritus or flushing may occur, whereas cutaneous mastocytosis may be associated with systemic mast cell mediator-related symptoms, including diffuse skin erythema, widespread blistering and, rarely, anaphylaxis.

Although the normal inflammatory markers, negative skin swabs and subsequent clinical course argued against infection, this was apparent only retrospectively. Empiric parenteral antibiotics and antivirals were therefore appropriate given the vulnerability of this age group to severe infection. The typical fragile bullae of bullous impetigo were absent, with the lesion instead developing a peau d'orange surface with crusted vesicles. Its initial uniform violaceous appearance raised the possibility of congenital haemangioma; however, evolution into a firm yellow-tan plaque made a vascular lesion less likely. JXG may similarly present as a solitary yellow lesion in infancy but is histologically distinct.

Histologically, mastocytomas show a dense dermal infiltrate of mast cells; however, typical mast cells may be difficult to recognize in neonatal lesions because of immature morphology or prior degranulation. Immunohistochemistry can therefore be valuable in confirming the diagnosis. CD117 (c-KIT) is a type I transmembrane tyrosine kinase receptor that binds stem cell factor, a critical regulator of mast cell proliferation, differentiation, survival and function.^3^^,^^4^ In our case, strong CD117 positivity confirmed mast cell lineage and supported the diagnosis.^2^^,^^3^

Given the isolated cutaneous disease, normal serum tryptase and absence of systemic features, KIT (D816 V) mutational analysis was not performed.^5^ Topical mometasone 0.1% was prescribed if further blistering occurred. Protective dressings were not required in this case but can be used to limit provocation of swelling and systemic symptoms.

Solitary cutaneous mastocytomas in infancy have an excellent prognosis, with most resolving spontaneously by puberty.^1^ This case highlights the importance of recognizing atypical neonatal presentations, which may facilitate earlier diagnosis and reduce unnecessary surgical intervention.

### Declaration of generative AI and AI-assisted technologies in the writing process

None.

## Conflicts of interest

None disclosed.
